# Bacterial Dynamics in Supraglacial Habitats of the Greenland Ice Sheet

**DOI:** 10.3389/fmicb.2019.01366

**Published:** 2019-07-03

**Authors:** Miranda Jane Nicholes, Christopher James Williamson, Martyn Tranter, Alexandra Holland, Ewa Poniecka, Marian Louise Yallop, The Black & Bloom Group, Martyn Tranter, Alexandre Anesio, Marian Yallop, Christopher Williamson, Ewa Poniecka, Miranda Nicholes, Alexandra Holland, Liane Benning, Jim McQuaid, Stefanie Lutz, Jenine McCutcheon, Andy Hodson, Edward Hanna, Tristam Irvine-Fynn, Joseph Cook, Jonathan Bamber, Andrew Tedstone, Jason Box, Marek Stibal, Alexandre Anesio

**Affiliations:** ^1^ Bristol Glaciology Centre, School of Geographical Sciences, University of Bristol, Bristol, United Kingdom; ^2^ School of Biological Sciences, University of Bristol, Bristol, United Kingdom; ^3^ School of Earth and Ocean Sciences, Cardiff University, Cardiff, United Kingdom; ^4^ Department of Environmental Science, Aarhus University, Roskilde, Denmark

**Keywords:** Greenland, ice sheet, bacterial production, bacterial abundance, glacier algae

## Abstract

Current research into bacterial dynamics on the Greenland Ice Sheet (GrIS) is biased toward cryoconite holes, despite this habitat covering less than 8% of the ablation (melt) zone surface. In contrast, the expansive surface ice, which supports wide-spread Streptophyte micro-algal blooms thought to enhance surface melt, has been relatively neglected. This study aims to understand variability in bacterial abundance and production across an ablation season on the GrIS, in relation to micro-algal bloom dynamics. Bacterial abundance reached 3.3 ± 0.3 × 10^5^ cells ml^−1^ in surface ice and was significantly linearly related to algal abundances during the middle and late ablation periods (*R*^2^ = 0.62, *p* < 0.05; *R*^2^ = 0.78, *p* < 0.001). Bacterial production (BP) of 0.03–0.6 μg C L^−1^ h^−1^ was observed in surface ice and increased in concert with glacier algal abundances, indicating that heterotrophic bacteria consume algal-derived dissolved organic carbon. However, BP remained at least 28 times lower than net primary production, indicating inefficient carbon cycling by heterotrophic bacteria and net accumulation of carbon in surface ice throughout the ablation season. Across the supraglacial environment, cryoconite sediment BP was at least four times greater than surface ice, confirming that cryoconite holes are the true “hot spots” of heterotrophic bacterial activity.

## Introduction

Bacteria play a key role in carbon cycling within every ecosystem on earth and the surfaces of ice sheets are no exception (Stibal et al., 2012a,b). The Greenland Ice Sheet (GrIS) is the largest permanent area of ice in the Northern Hemisphere and the most expansive supraglacial ecosystem on Earth (Mernild et al., 2010). Once considered devoid of life, high surface melt on the GrIS provides habitats for diverse and active prokaryote and eukaryote communities, which strongly influence both physical (melt) and chemical (carbon and nutrient cycling) surface characteristics (Nghiem et al., 2012; Anesio et al., 2017). In particular, heterotrophic bacterial communities impact carbon cycling on both local and regional scales *via* the remineralization of organic carbon (Stibal et al., 2008; Anesio et al., 2010; Cook et al., 2012; Telling et al., 2012).

To date, studies investigating bacterial abundance and production from the surface of the GrIS have predominantly focused on snow-pack environments and cryoconite holes; water-filled depressions in the ice surface formed by preferential melt-in of dark organic and inorganic particles (Hodson et al., 2007, 2010; Anesio et al., 2010; Cook et al., 2012; Stibal et al., 2012b; Chandler et al., 2015). Cryoconite holes have been identified as “hot spots” of carbon cycling, in which heterotrophic bacteria, at abundances ranging 10^6^–10^9^ cells g^−1^ sediment, play a key role (Anesio et al., 2010). Autotrophs (predominantly cyanobacteria) produce dissolved organic carbon (DOC), which is assimilated by heterotrophic bacteria and converted to biomass, hereafter referred to as bacterial production (BP; Ducklow, 2000; Kirchman, 2001). Carbon is either respired (remineralized to carbon dioxide) or retained within bacterial cells and transferred up the food chain *via* grazing (Ducklow, 2000; Pomeroy et al., 2007). Carbon fluxes through cryoconite holes are well defined within the literature and indicate that carbon cycling in the supraglacial environment occurs on globally significant scales (Hodson et al., 2007, 2010; Cook et al., 2012).

In comparison to cryoconite holes, which constitute 0–8% of the GrIS supraglacial environment (Cook et al., 2012), bacterial dynamics in the expansive surface ice remains relatively understudied (Hodson et al., 2015), and therefore largely neglected within estimates of carbon cycling. Recently, research has focused on blooms of Streptophyte microalgae (hereafter “glacier algae”), which occur in surface ice during summer ablation periods. These highly pigmented cells, often mistaken for dust or impurities, drive reductions in ice surface albedo (reflectance) (Yallop et al., 2012; Lutz et al., 2014; Stibal et al., 2017) with significant implications for accelerating melt (Tedstone et al., 2017; van den Broeke et al., 2017; Ryan et al., 2018). Bacterial abundance in surface ice has been shown to range 1.9–28 × 10^3^ cells ml^−1^ (Stibal et al., 2015); however, only one study has quantified bacterial production within this habitat, indicating rates of BP were 30-times less than net primary production of supraglacial glacier algal communities (Yallop et al., 2012). Thus far, no integrated study has determined both bacterial abundance and production within the surface ice environment, or the relationship between bacterial and glacier algal communities throughout summer ablation seasons. Understanding the role of bacterial communities in one of the most expansive habitats on the GrIS surface is fundamental to accurately determine the flux of carbon through every habitat of the supraglacial environment.

To this end, the aim of the present study was to investigate bacterial abundance and production in the surface ice on the south-western GrIS, throughout summer ablation seasons, and in relation to glacier algal bloom dynamics. Our data provide the first integrated assessment of heterotrophic bacterial production across the surface ice environment and demonstrate the relative contributions of different supraglacial habitats to the wider carbon cycling on the surface of the GrIS.

## Materials And Methods

### Study Area

**Figure 1:**
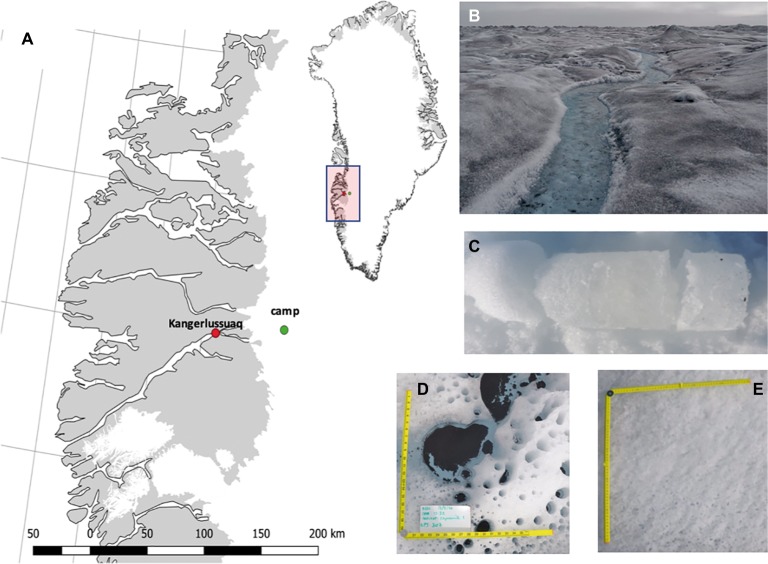
A map of Greenland highlighting the camp at which sampling was conducted **(A)**; images of surface ice with visible algal bloom **(B)**; an example ice core **(C)**; cryoconite holes **(D)**; clean surface ice with no algal bloom **(E)**.

Sampling of surface ice habitats of the Greenland Ice Sheet (GrIS) to determine bacterial abundance and production was performed during July and August 2016 and June 2017. This allowed us the capture bacterial dynamics at different growth phases of the algal bloom during the relatively short ablation season (Table 1). All sampling and incubations were performed at a primary ice camp, established within the GrIS ablation zone approximately 35 km from the south-western ice sheet margin (67° 04′43.3″ N, 49° 20′29.7″ W), adjacent to the S6 PROMICE weather station (Figure 1).

**Table 1:** Sampling dates for bacterial abundance and production of each habitat categorized by time as pre- snowline retreat (i.e., snow covered the surface ice), early ablation period (June 2017), mid ablation period (July 2016), and late ablation period (August 2016).

Ablation period	Habitat	Sampling date
Pre-snow line retreat	Snow pit	02.06.17
Early ablation season	Surface ice	13.06.17 24.06.17
	Ice core	11.06.17 22.06.17
Mid ablation season	Cryoconite sediment	16.07.16 22.07.16
	Surface ice	16.07.16
Late ablation season	Cryoconite sediment	31.07.16 15.08.16
	Surface ice	15.08.16

### Sampling Procedure

To examine bacterial dynamics prior to the retreat of the snowline, *n* = 3 snow pits were dug at the primary ice camp location using a clean metal shovel, and discrete snow pack layers (i.e., snow layers separated by superimposed ice) transferred into sterile Whirl-Pak bags. During the early ablation season, i.e., shortly after the retreat of the snowline (see Table 1), 1 m deep ice cores (*n* = 2), were sampled using a KOVACS ice corer (Roseburg, USA). Ice cores were sectioned using a clean hand saw into visually discrete layers (e.g., weathering crust, previous years cryoconite layer, dense sub-surface ice, see Figure 1) and placed into sterile Whirl-Pak bags for melting. Given that snow line retreat resulted in a highly heterogeneous surface that included snow patches, clean bare ice, and ice supporting algal blooms, samples were taken at random across surface ice to capture bacterial dynamics across this heterogeneity during the early ablation period (*n* = 6). In contrast, during the middle and late ablation periods, surface ice was categorized as following Yallop et al. (2012) and Williamson et al. (2018). Glacier algae are highly pigmented and darken the surface ice (Figure 1; Yallop et al., 2012; Williamson et al., 2018); therefore, depending on the visual darkness of ice, 1m^2^ sections were categorized as containing a low, medium, or high coverage of glacier algae (Figure 1; Table 1). Triplicates of surface ice of each algal coverage were sampled by using a clean ice saw to remove the top 2 cm of ice (approximately 1,068 ± 364 ml of ice), which was subsequently melted at ambient temperatures in the dark in over ~ 24-h period. Sampling tools were washed between samples with Milli-Q water. Finally, to contrast bacterial abundance and production between surface ice environments and the better studied cryoconite holes, cryoconite sediment was sampled in triplicate from cryoconite holes on four occasions across the mid-late ablation period (Table 1), with sediment transferred into sterile Whirl-Pak bags using a large sterile pipette.

### Bacterial and Algal Cell Enumeration

To enumerate bacterial abundance, 15 ml subsamples were taken from all melted snow and ice samples following homogenization and fixed with 25% glutaraldehyde at 2% final concentration. For cryoconite sediment, a small subsample was removed and placed with 1 ml of Milli-Q water into a 2 ml Eppendorf tube, and fixed with glutaraldehyde as above. All fixed snow, ice, and cryoconite sediment samples were transported back to the University of Bristol under chilled conditions. Bacterial abundance was determined from 1 ml of snow and ice samples or approximately 100 mg (wet mass) of cryoconite sediment by epifluorescence microscopy following staining with 4′, 6-diamidino-2-phenylindole (DAPI, Sigma) at a final concentration of 10 μg ml^−1^ (Porter and Feig, 1980). Prior to staining, samples were vortexed for 10 s and sonicated at 30°C for 1 min to facilitate cell detachment from particles (Duhamel and Jacquet, 2006). The staining, filtering, and mounting procedure was conducted as outlined by Bradley et al. (2016). Bacterial cells were counted using an Olympus BX41 microscope at 1,000× magnification. A minimum of 300 cells or 30 randomly selected grids (each 10^4^ μm^2^) were counted. For all snow and ice samples, enumeration of glacier algal abundance was performed using a Fuchs-Rosenthal hemocytometer (Lancing, UK) on a Leica DM 2000 epifluorescence microscope.

### Bacterial Production

#### Incubation

Bacterial production within all snow, ice, and cryoconite sediment samples was estimated by the incorporation of ^3^H-leucine using the microcentrifuge method (Kirchman, 2001). For snow and ice cores and surface ice samples, 1.5 ml of melted sample was added to a 2 ml sterile microcentrifuge tube and incubated in triplicate *in vitro* on the ice sheet under the same environmental conditions as in the habitat of origin for 3 h ± 20 min. For cryoconite sediment samples, ~100 mg wet weight sediment and 1 ml water were incubated in 2 ml sterile microcentrifuge tubes. Incubations were initiated by addition of ^3^H-leucine at a final concentration of 40 nM, and terminated by the addition of 90 μl 100% trichloroacetic acid (TCA).

#### Processing

Samples were transported back to the University of Bristol and processed after Kirchman (2001). All samples were centrifuged at 16,000× *g* for 20 min and the supernatant aspirated. Each sample was washed twice, first with 1.5 ml of 100% TCA followed by 1.5 ml of 80% ethanol. After washing, samples were left for 24 h to allow the ethanol to evaporate. Cryoconite sediment samples were weighed to determine the dry weight of the sediment used in the incubation for normalization of rates. All samples were analyzed by liquid scintillation counting (Beckman LS 6000 IC, Beckman Instruments, Fullerton, CA, USA). Disintegrations per minute (DPM) were corrected using a linear relationship between sediment weight of cryoconite and the DPM.

#### Data Processing

To estimate bacterial production, the DPM from killed controls were subtracted from live samples and converted to bacterial production (BP) after Smith and Azam (1992), assuming an isotope dilution of two (Simon and Azam, 1989). BP for surface ice samples were converted from units of volume (L^−1^) to area (m^−2^) after Williamson et al. (2018). Briefly, the top 2 cm of ice was removed, as outlined previously, from one meter squared of ice (5 replicates) with varying algal coverage and melted in WhirlPak bags. A graduated cylinder was used to measure 1,068 ± 364 ml of water per m^2^ of surface ice sampled. Similarly, bacterial production in cryoconite sediment (g^−1^) was converted to units of area (m^−2^) using mean cryoconite mass across a square meter (255 g m^−2^) from a study site at a similar distance from the ice margin as our primary field camp, reported by Cook et al. (2012).

### Data Analysis

All analyses and plotting of data were performed using R v.3.4.1 (R Core Team, 2017). Levene’s test for Homogeneity of Variance and Shapiro-Wilks Normality Test were performed on all data prior to the application of parametric analyses. Parametric assumptions were not met for data across all ablation periods; however, assumptions were valid when bacterial production was analyzed per ablation period. Consequently, one-way Analysis of Variance (ANOVA) was conducted in relation to algal coverage (three levels) for each ablation period. *Post Hoc* Tukey HSD was performed on all significant ANOVA analyses. Least squares linear regression was applied to examine the relationships between bacterial and algal abundance, and bacterial production.

## Results

### Bacterial Abundance

**Figure 2:**
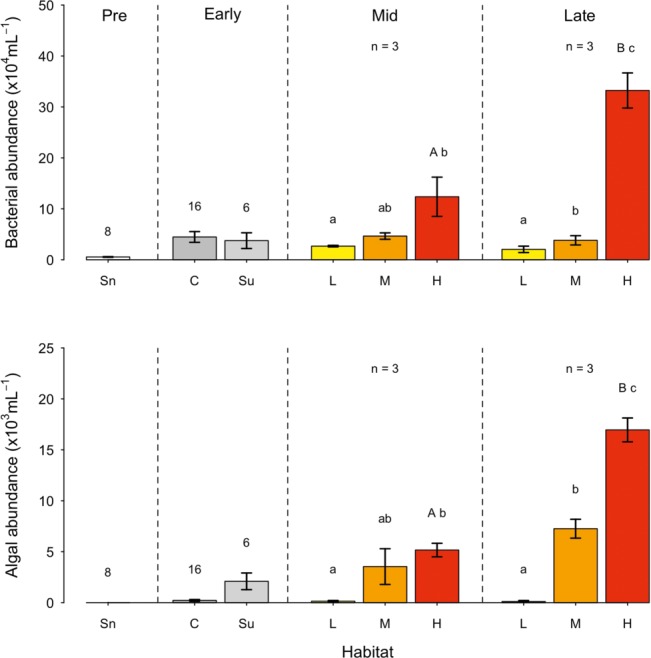
Bacterial and algal abundance (mean ± SE) across the ablation season (sectioned as Pre: Pre-snowline retreat; early, mid, and late ablation periods) in snow (Sn); Ice cores (C); Surface ice (Su); and surface ice with a low (L), medium (M), and high (H) algal coverage. Numbers above bars represent the *n* of the sample. Letters above bars represent homogenous groups determined by a one-way ANOVA (abundance ~ algal coverage) and capital letter denote homogenous group determine by a *t*-test between surface ice with a high algal coverage between mid and late ablation periods.

Bacterial abundance in the snowpack environment (prior to the retreat of the snowline) averaged 5.5 ± 0.6 × 10^3^ cells ml^−1^ (Figure 2), with no significant difference in bacterial abundance between snow pack layers (i.e., snow layers separated by superimposed ice) evident. In contrast, bacterial abundance was 10-fold greater in surface ice and ice core samples during the early ablation period. While no significant difference in bacterial abundance was apparent between different ice core layers, higher abundances were generally associated with core layers containing cryoconite sediment from previous ablation seasons. During the mid and late ablation periods, bacterial abundance varied in concert with algal coverage on the surface ice, whereby significantly higher bacterial abundance was apparent in ice with a high algal coverage (mid: *F*_8_ = 5.16, *p* < 0.05; late: *F*_8_ = 70.39, *p* < 0.01). From the mid to late ablation period, bacterial abundance increased significantly in ice with a high algal coverage (*t*_5_ = −4.04, *p* < 0.05), reaching an overall maximum of 3.3 ± 0.3 × 10^5^ cells ml^−1^ by the end of the ablation period.

### Glacier Algal Abundance

Comparative monitoring of glacier algal assemblages demonstrated the presence of two main species, *Ancylonema nordenskiöldii* and *Mesotaenium berggrenii,* consistent with the findings of Williamson et al. (2018) and Lutz et al. (2018). While glacier algal cells were absent from all snowpack samples (Figure 2) an average of 2.2 ± 0.8 × 10^2^ cells ml^−1^ were found in ice cores, with no significant difference in glacier algal abundance between different core sections. In the exposed bare ice surface during the early ablation period, glacier algal abundance was an order of magnitude greater than abundance in ice cores, averaging 2.1 ± 0.8 × 10^3^ cells ml^−1^. As with bacterial abundance, glacier algal abundance was significantly different between ice surfaces visually categorized as containing a low, medium or high algal coverage (mid: *F*_8_ = 5.6, *p* < 0.05; late: *F*_8_ = 95.5, *p* < 0.01; Figure 2). The highest glacier algal abundance of 1.7 ± 0.1 × 10^4^ cells ml^−1^ was observed at the end of the ablation period, with a significant increase in glacier algal abundance apparent from the mid to late ablation period (*t*_5_ = −8.74, *p* < 0.01). No significant relationship was apparent between glacier algal and bacterial abundance in snow pack, surface ice, or sub-surface ice environments prior to snow-line retreat or during the early ablation period. However, during both the mid and late ablation periods, significant linear relationships were apparent between glacier algal and bacteria abundance (*R*^2^ = 0.62, *p* < 0.05; *R*^2^ = 0.78, *p* < 0.001, respectively, *n* = 9 in both cases). As a comparison to the ice surface, bacterial abundance was determined within cryoconite sediment. Abundances were found to average 3.0 ± 1.0 × 10^7^ cells g^−1^ (dry weight) during the mid-ablation period and 1.8 ± 0.3 × 10^7^ cells g^−1^ (dry weight) during the late ablation period. Unlike the ice surface, there was no significant change in bacterial abundance in cryoconite across the ablation season.

### Bacterial Production

**Figure 3:**
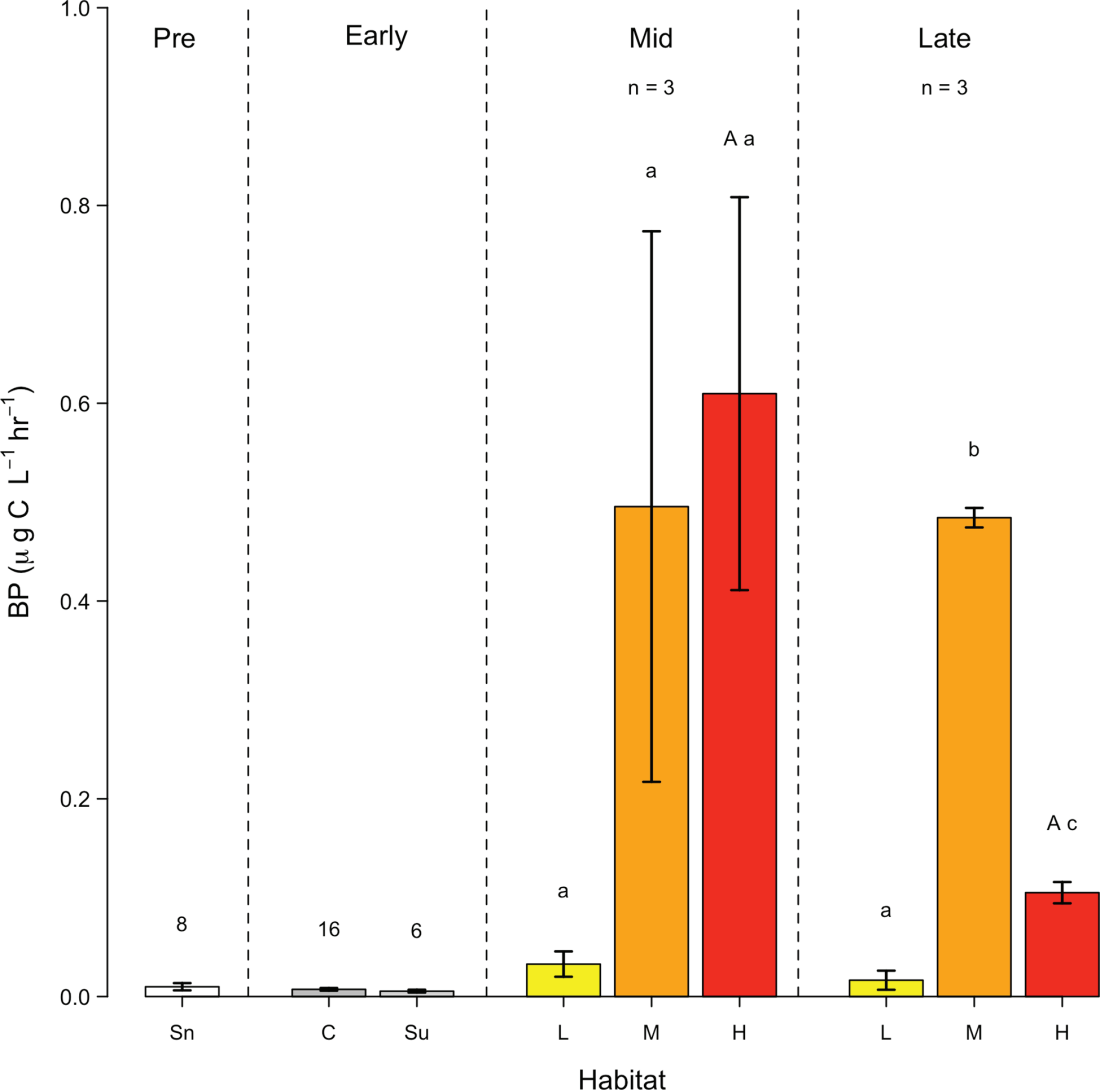
Bacterial production (BP), (mean ± SE) across the ablation season (sectioned as Pre: Pre-snowline retreat; Early, Mid and Late ablation periods) in snow (Sn); Ice cores (C); Surface ice (Su); and surface ice with a low (L), medium (M) and high (H) algal coverage. Numbers above bars represent the *n* of the sample. Lower-case letters above bars represent homogenous groups determined by a one-way ANOVA (abundance ~ algal coverage) and a capital letter denote homogenous group determine by a *t*-test between surface ice with a high algal coverage between mid and late ablation periods.

Bacterial production (BP) was negligible (i.e., less than 0.04 μg C L^−1^ h^−1^) in snow pack, surface ice, and sub-surface ice sampled prior to snow line retreat and during the early ablation period (Figure 3), reflecting the low bacterial abundance apparent. During the mid-ablation period, bacterial production ranged from 0.03–0.6 μg C L^−1^ h^−1^. While BP was generally increased in ice with a medium or high algal coverage, as compared to a low cover, these differences were not statistically significant, and no significant relationship was apparent between BP and bacterial or glacier algal abundance during the mid-ablation period. In contrast, there was a significant difference in BP between ice containing a low, medium or high algal coverage during the late ablation period (*F*_8_ = 603.4, *p* < 0.01 between different algal coverage), with maximal BP apparent in areas of medium algal coverage (0.50 ± 0.12 μg C L^−1^ h^−1^). In contrast to bacterial abundance, no significant change in BP was observed in surface ice with high algal coverage from the mid to late ablation period, with cell normalized rates of activity (data not shown) decreasing by approximately 96% over this period.

Bacterial production in cryoconite sediment was negligible (< 0.01 μg C g^−1^ h^−1^) during the mid-ablation period, with a significant increase (*F*_5_ = 8.061, *p* < 0.05) to 0.02 ± 0.002 μg C g^−1^ h^−1^ apparent during the late period. At the cellular level, BP in cryoconite sediment increased from an average of 1.0 ± 0.5 fg C cell^−1^ h^−1^ during mid-ablation to 2.8 ± 0.3 fg C cell^−1^ h^−1^ by the end of the ablation period. Cryoconite sediment accounts for approximately 2.30 ± 0.80 and 5.1 ± 0.51 μg C m^−2^ h^−1^ of bacterial production across the ice sheet surface during the mid and late ablation period respectively, representing at least a ~ four-fold greater BP in cryoconite sediment than in surface ice containing a high algal coverage.

## Discussion

Previous research into heterotrophic bacterial dynamics within the supraglacial landscape has predominantly focused on cryoconite holes as opposed to the more expansive surface ice environment. This study provides the first integrated assessment of bacterial abundance and production in surface ice of the south-western Greenland Ice Sheet (GrIS), demonstrating the development of bacterial communities from pre-snow line retreat, to the end of the ablation period, in tandem with glacier algal bloom monitoring. Overall, our data demonstrate that bacteria in the surface ice are likely consuming organic carbon produced by blooms of glacier algae; however, carbon cycling by heterotrophic bacteria in surface ice is significantly less efficient than in cryoconite sediment.

At the start of the ablation season, snow samples contained bacteria at abundances comparable with those previously reported for snow from the south western GrIS (10^2^–10^3^ cells ml^−1^; Cameron et al., 2015). Bacteria in snow can originate from soil particles and marine aerosols deposited by the wind, which are transported from predominantly local terrestrial, marine and glaciated regions (Cameron et al., 2015; Šantl-Temkiv et al., 2018). Genetic similarities between bacterial communities in snow and surface ice were previously identified by Musilova et al. (2015), who inferred that the snowpack likely inoculated the supraglacial environment. The bacterial abundances in ice cores demonstrated here, particularly in layers containing cryoconite particles, suggests a retention of bacterial cells in shallow sub-surface ice from the previous year’s ablation seasons that likely also seed surface ice environments with the onset of melt. In contrast, an absence of glacier algae from snow pack environments at the start of the ablation season suggests that, unlike bacteria, aeolian deposition is not likely an important source of cells. Currently, very little is known about the initiation of supraglacial glacier algal blooms on the Greenland Ice Sheet, including the source of cells. We demonstrate here the presence of glacier algae within shallow sub-surface ice cores at the very start of the ablation period, suggesting the retention of glacier algal cells between ablation seasons. Meltwater flushing of these cells to the ice surface may serve to initiate glacier algal blooms with the onset of melt, though further research is required to confirm this assertion.

Bacterial abundance in surface ice during the middle of the ablation season was 10–100 times greater than abundances identified by Stibal et al. (2015) from sites within the same region of the GrIS. By deliberately sampling surface ice visually containing a range of algal coverage, we were able to demonstrate that substantial heterogeneity is apparent in bacterial abundance across supraglacial environments. Bacterial communities in supraglacial environments of the GrIS predominantly comprise of Actinobacteria, Proteobacteria, and Bacteroidetes (Musilova et al., 2015; Cameron et al., 2016; Smith et al., 2018; Perini et al., 2019). These bacteria rely on external sources of organic carbon which, in glacial landscapes, typically originate from a variety of allochthonous (terrestrial) and autochthonous (*in situ* photosynthesis) sources, with varying levels of bioavailability (Hood et al., 2009; Bhatia et al., 2010; Lawson et al., 2014; Antony et al., 2017). Inputs of allochthonous carbon decrease with distance from the ice sheet margin (Stibal et al., 2012a); therefore, organic carbon at our study site is likely sourced from *in situ* photosynthesis. Glacier algae residing within surface ice have been shown to actively fix carbon (Yallop et al., 2012; Williamson et al., 2018) and, although no studies have been conducted on the quantity of dissolved organic carbon (DOC) exuded from glacier algae, it has been found that 20–50% of carbon fixed by microalgae can be released as DOC (Zlotnik and Dubinsky, 1989; Hulatt and Thomas, 2010). It is likely that such DOC sources play a significant role in supporting the BP observed here, particularly during the middle of the ablation season. Carbon is incorporated into bacterial biomass, stimulating growth, and cell division, results in the highly significant linear relationship identified here between algae and bacterial abundance.

During the mid-ablation period, bacterial production in surface ice was found to be substantially lower than glacier algae primary production. Surface ice with a high algal coverage had a net primary production (NPP) 35 times higher than BP (Table 2; Williamson et al., 2018), likely indicating that more organic carbon is produced by glacier algae than is consumed by heterotrophic bacteria. It is possible that bacteria in surface ice, particularly those deposited by the wind, may not have necessary adaptations required to thrive in this extreme environment (Smith et al., 2018). For example, some species of bacteria may not produce photo-protective pigments; therefore, unlike glacier algae, the cell is exposed to harmful levels of radiation (Ravanat et al., 2001; Remias et al., 2012a,b). Intense radiation may also influence glacier algal-derived DOC through photochemical transformations (Antony et al., 2018). In freshwater environments, UV radiation has been shown to either inhibit BP, through the production of hydrogen peroxide (Tranvik and Kokalj, 1998) and recalcitrant DOC compounds or stimulate BP by degrading large compounds into more bioavailable species (Lindell et al., 1995; Bertilsson and Tranvik, 2000; Amado et al., 2015). It is therefore possible that algal-derived DOC is made unavailable to heterotrophic consumption through photochemical alteration, though further research is required to confirm this hypothesis.

**Table 2:** The net primary production (NPP; sourced from Williamson et al., 2018) and the ratio of NPP to bacterial production (BP) from surface ice containing low, medium and high algal coverage for the mid and late ablation periods.

Algal coverage	Net primary production (mg C L^−1^ day^−1^)	Ratio of NPP to BP	
		Mid	Late
Low	~0.16	202:1	404:1
Medium	~0.32	27:1	28:1
High	~0.52	35:1	260:1

Consistent with the mid-ablation period, the highest abundances of bacteria were observed in areas with high algal coverage during the late ablation period. However, BP in surface ice with a high algal coverage was substantially lower than during the mid-ablation period with rates of NPP 260 times greater than BP (Table 2). We suggest that this may reflect nutrient limitation and competition between microbial communities. During an algal bloom, the sheer biomass of algal cells likely intercepts and retains the majority of available nutrients resulting in severe nutrient limitation for bacteria, particularly toward the end of the season, where inputs from snowmelt are reduced (Holland et al., in press). Nutrient limited conditions may also alter the quality and composition of DOC excreted by glacier algae (Dodds, 2010) resulting in less bioavailable species and a subsequent reduction in BP.

Bacterial production measured in cryoconite sediment was found to be lower than BP previously recorded on other Arctic glaciers (Table 3). This likely represents the distance of the study site from the ice sheet margin and a lower input of allochthonous carbon compared to valley glaciers (Stibal et al., 2012a). Bacteria in cryoconite holes consume DOC fixed by cyanobacteria (Anesio et al., 2009), with BP increasing significantly from the mid to late ablation period. It is possible that during the mid-ablation period, organic carbon is retained in surface ice along with glacier algae cells and particles; however, during the late period, it may be washed into cryoconite holes, supplementing DOC produced by cyanobacteria sustaining higher BP.

**Table 3:** Comparison of bacterial production (BP) in cryoconite sediment from Arctic glaciers measured using leucine incorporation.

Location	BP (ng C g^−1^ h^−1^)		References
South West Greenland	Mid ablation period: 9.0 ± 3.0	Late ablation period: 20 ± 2.0	This study
Midtre Lovénbreen, Svalbard	40 ± 19		Hodson et al., 2007
Midtre Lovénbreen, Svalbard	50 ± 10		Bellas et al., 2013
Austre Brogerbreen, Svalbard	50 ± 10		
Russell Glacier, Greenland	70 ± 1.0		
	60 ± 10		
Austre Brogerbreen, Svalbard	9.0 ± 6.0		Anesio et al., 2010
Midtre Lovénbreen, Svalbard	40 ± 18		

Our estimates of BP across a representative square meter of the supraglacial environment indicate that bacterial heterotrophic production in cryoconite holes is four times greater than in the surface ice, despite these habitats accounting for less than 8% of the GrIS ablation zone (Cook et al., 2012). Bacteria in cryoconite sediment are protected from extreme levels of UV radiation, temperature, and pH fluctuations, which allow the development of a functionally diverse microbial community driving an efficient microbial loop (Musilova et al., 2015). Additionally, bacteria capable of nitrogen fixation (Telling et al., 2011) and phosphorus extraction from mineral particles (Stibal et al., 2009) supply additional inorganic nutrients and reduce nutrient limited conditions, compared to the ice surface. These factors result in heterotrophic carbon cycling exceeding that on the ice surface, confirming that cryoconite holes are truly “hot spots” of heterotrophic activity (Anesio et al., 2009).

## Conclusions

Despite the potential influence on carbon cycling within the supraglacial environment, bacterial abundance, and activity within surface ice on the Greenland Ice Sheet has been largely understudied by the scientific community. This study aimed to identify spatial and temporal trends in the abundance and activity of heterotrophic bacteria within the supraglacial environment. Our results indicate that bacteria are spatially heterogeneous across the surface ice environment with influxes of cells from aeolian deposition as well as melting surface ice. The significant linear relationship between bacterial and algal abundances suggests organic carbon produced by glacier algae is being consumed; however, our study indicates that net primary production significantly exceeds bacterial production. This suggests that the remineralization of carbon by heterotrophic bacteria is inefficient on the surface ice, compared to cryoconite holes, and organic carbon accumulates within the surface ice. The fate of this organic carbon is currently unclear; however, several studies indicate extensive carbon export from ice sheets, which could stimulate bacterial production in downstream environments (Bhatia et al., 2013; Hawkings et al., 2015; Musilova et al., 2017).

## Data Availability

The datasets generated for this study are available on request to the corresponding author.

## Author Contributions

MN, CW, AA, and MY conceived and designed the study. CW, AA, MT, EP, AH, and the Black and Bloom group collected samples and conducted leucine incubations on the ice sheet. CW provided algal counts for the mid and late ablation periods. MN conducted bacterial counts and was aided by CW with leucine analysis. MN wrote the paper with inputs from CW, AA, and MY, and all authors reviewed the final manuscript.

### Conflict of Interest Statement

The authors declare that the research was conducted in the absence of any commercial or financial relationships that could be construed as a potential conflict of interest.
